# Maize Dwarf Mosaic Virus: From Genome to Disease Management

**DOI:** 10.3390/v10090492

**Published:** 2018-09-13

**Authors:** Maathavi Kannan, Ismanizan Ismail, Hamidun Bunawan

**Affiliations:** 1 Institute of Systems Biology, Universiti Kebangsaan Malaysia, 43600 Bangi, Malaysia; maathavikannan95@gmail.com (M.K.); maniz@ukm.edu.my (I.I.); 2 School of Bioscience and Biotechnology, Faculty of Science and Technology, Universiti Kebangsaan Malaysia, 43600 Bangi, Malaysia

**Keywords:** *Maize dwarf mosaic virus*, genome, transmission, symptomatology, diagnosis, management

## Abstract

*Maize dwarf mosaic virus* (MDMV) is a serious maize pathogen, epidemic worldwide, and one of the most common virus diseases for monocotyledonous plants, causing up to 70% loss in corn yield globally since 1960. MDMV belongs to the genus Potyvirus (*Potyviridae*) and was first identified in 1964 in Illinois in corn and Johnsongrass. MDMV is a single stranded positive sense RNA virus and is transmitted in a non-persistent manner by several aphid species. MDMV is amongst the most important virus diseases in maize worldwide. This review will discuss its genome, transmission, symptomatology, diagnosis and management. Particular emphasis will be given to the current state of knowledge on the diagnosis and control of MDMV, due to its importance in reducing the impact of maize dwarf mosaic disease, to produce an enhanced quality and quantity of maize.

## 1. Introduction

Maize (*Zea mays* L.) is one of the most highly cultivated crops worldwide. It is the third largest crop grown in the developing world and it has been identified as a major staple food in Africa [1]. In south and eastern Africa, maize serves as a primary food for more than 24 million families and also as a subsistence food source for poor populations outside Asia [2]. Approximately 177 million hectares of tropical, semitropical and temperate zones are used to cultivate maize [3] and 875 million metric tons of maize are produced every year [4].

More than 50 viruses have been shown to infect maize [5]. These include *Sorghum mosaic virus* (SrMV) [6,7], *Johnsongrass mosaic virus* (JGMV) [8], *Maize chlorotic mottle virus* (MCMV), and *Maize chlorotic dwarf virus* (MCDV) [9]. Among all the maize-infecting viruses, *Maize dwarf mosaic virus* (MDMV) is the most common disease agent in this monocotyledonous crop globally [10], with incidences of maize dwarf mosaic (MDM) disease reported in Africa, United States, Asia and Europe [11].

MDMV strains’ nomenclature was in a state of confusion, since MDMV is intimately correlated with the *Sugarcane mosaic virus* (SCMV) [12]. The organization of inclusions and alterations produced by four isolates representing different virus strains from the subgroup of SCMV in infected cells were studied and differentiated by cellular pathology [13]. The results obtained demonstrated the presence of typical inclusions only in MDMV infected cells, but laminar aggregations were also exist in SCMV infected cells [14].

MDMV is classified in *Potyviridae*, the most numerous group of plant viruses [15]. There are 8 genera under the family; *Potyvirus*, *Tritimovirus*, *Brambyvirus*, *Rymovirus*, *Ipomovirus*, *Bymovirus*, *Macluravirus*, and *Poacevirus* [16]. MDMV belongs to the genus *Potyvirus*. In 1964, flexuous rod-shaped MDMV with a length of 750 nm and diameter of 12–15 nm was identified for the first time in Illinois [17]. MDMV is a holoparasite that requires a vector or host to reproduce and survive [18]. The infected plants take up to 15 days to exhibit the symptoms [19]. In this case, both serological and molecular methods have been developed to diagnose the MDM diseased plants more efficiently, eliminating the dependence on symptoms observation for identification purposes. The viral particles were observed to be distributed generally in cytoplasm and infrequently in plasmodesmata [20]. The lowest temperature needed to inactivate MDMV completely is 54–58 °C. At room temperature, survival in vitro for MDMV lasts 1–2 days while at 0–4 °C, it lasts 3–5 days [21,22].

In general, the loss in maize yield caused by MDMV can be up to 70% [23,24], mainly due to a reduction in the rate of photosynthesis and elevation in the rate of respiration [25]. Taking into account the economic significance of the disease, our current review has discussed available information from genome to global management of MDMV, so that greater effort would be put towards further research work in controlling MDM disease for the maintenance of profitable production and the good health of maize crops in future.

## 2. Genome

Like other potyviruses, MDMV is a positive-stranded RNA [18,26]. The MDMV genome is ~9500 base pairs in length with a covalently bounded viral-genome-linked protein, VPg at its 5′ end as well as a poly (A) tail attached to its 3′ end [10]. A large 338 kDa polyprotein is translated from a single open reading frame (ORF) [27], which is subsequently proteolytically cleaved by three self-coded proteinases to yield 10 final proteins (P1, HC-Pro, P3, 6K1, CI, 6K2, NIa-VPg, NIa-Pro, NIb, and CP) with multiple functions [28,29]. The last cistron that encodes the capsid protein (CP) [30] is greatly conserved among numerous potyvirus species [31]. This protein subunit of MDMV has a molecular weight of 28.5 × 10^3^ according to amino acid analyses [32]. The C-terminal regions of the coat protein primarily plays a role in the process of encapsidation and cell-to-cell transport and the flexible N-terminus is involved in long distance and systemic transport and contains the DAG motif essential for aphid transmission competence [33].

Several complete sequences of MDMV isolates are currently available (Table 1). The viral genome of MDMV-BG is composed of 9515 nucleotides and contains an open reading frame encoding 3042 amino acids, flanked by 30 and 50 untranslated regions (UTRs) of 139 and 250 nucleotides, respectively. Meanwhile, the genome of MDMV-Sp is made up of 9414 nt and contains one ORF encoding 3042 amino acids, flanked by 50 and 30 UTRs of 138 and 234 nucleotides, respectively. The similarities in nucleotide and amino acid sequence between MDMV-BG and MDMV-Sp are 85% and 93%, respectively [27]. MDMV-OH1 encoded P1 protease (nt 1400–838), HC-Pro (nt 839–2218), P3 protease (nt 2219–3259), 6K1 (nt 3260–3460), CI (nt 3461–5350), 6K2 (nt 5351–5509), NIa-VPg (nt 5510–6076), NIa-Pro (nt 6077–6802), NIb-replicase (nt 6803–8365), CP (nt 8366–9180). It also encodes the “PIPO” (nt 2678-2922) that overlaps the P3 gene. PIPO is a new ORF recently described to be expressed as a result from the transcriptional slippage specific to the viral RNA polymerase [34], it well conserved and has a strong bioinformatics coding signature throughout the *Potyviridae* family members [35]. It is translated in the +2 reading frame relative to the potyviral long ORF as the P3N-PIPO fusion protein [36]. Potyviral P3N-PIPO interacts with the PCaP1 host plasma membrane protein to function in cell-to-cell movement [37]. MDMV-OH1 has an identity of about 97% and 98% in nucleotide sequences and polyprotein amino acid sequences respectively with MDMV-OH2. Both MDMV-OH isolates are more related to MDMV-It isolate rather to other European isolates based on the phylogenetic tree constructed.

The construction of the full-length infectious cDNA clones has been an important technique in the understanding of virus fundamental mechanisms studies [42]. Moreover, generation of infectious clone allows mutagenesis studies, insertion, substitution and deletions, to be conducted at any particular region of the viral genome [43]. Gell [44] initiated the attempt to develop the full-length virulent MDMV clones. Unfortunately, no typical symptoms of MDMV infection were observed in the inoculated plants and no viral particles were detected using the polymerase chain reaction (PCR) approach. The next attempt was made in 2012, Stewart et al. [40] managed to produce the full-length infectious MDMV cDNA clone using MDMV-OH1 isolate. The study managed to overcome the toxicity exhibited by plasmids containing full-length MDMV in *Escherichia coli*. According to Ali et al. [45], cDNA clones are often unstable due to the toxicity of the viral proteins such as P3 and CI for bacteria.

In earlier literature, there was a considerable confusion in the taxonomy of the potyvirus, due to huge size of the group and wide diversification among the species [6]. MDMV strains have been differentiated serologically [46,47] and by host range [48,49]. Serological relationship investigation of 13 American and 4 Australian strains of MDMV and SCMV using virus-specific polyclonal antisera directed against N-terminal regions of their coat proteins led to the establishment of four groups of viruses: (1) MDMV (MDMV-A, MDMV-D, MDMV-E, MDMV-F); (2) SCMV (MDMV-B); (3) JGMV (MDMV-O) and (4) SrMV forming a subgroup of SCMV [50]. This shows that a serological approach has the potential to be utilized as a taxonomic parameter.

In the context of host range, of the two principal strains, MDMV-A was found to be a johnsongrass-infecting strain, while MDMV-B is a non-johnsongrass-infecting strain [51]. MDMV-A was further differentiated from MDMV-B by the means of relative molecular mass (Mr) of RNA. Mr of MDMV-A RNA derived from three methods showed a value of 3.3 × 10^6^ and for MDMV-B, it was reported as 3.0 × 10^6^ [52]. Comparison in RNA nucleotide composition showed that both MDMV-A and MDMV-B strains consist of adenylic and guanylic acids in same quantity. Nevertheless, their high adenylic content in general adds credence to the suggestion that high content of adenylic acid might be a characteristic of potyviruses [53]. Furthermore, MDMV-A has a higher cytidylic acid content but lower uridylic acid content than MDMV-B [32].

## 3. Transmission

**Figure 1:**
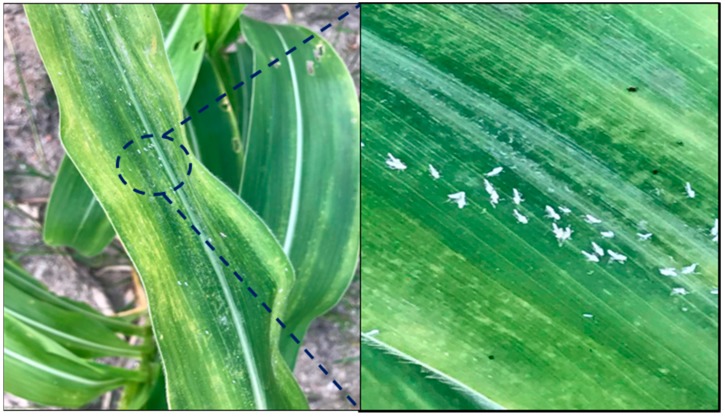
Mosaic symptoms on the lower leaves and yellow streaks along veins in corn plant as it approaches maturity. Also the fitch, *Rhopalosiphum maidis*, nymphs on the maize leaves.

Aphids are the most widely spread vectors of plant viruses and have the potential to transmit both persistent and non-persistent viruses. Aphids transmit more than 200 species of plant viruses in a non-persistent way [54]. Among them, insects of the order Homoptera, with their piercing-sucking mouthparts, are highly efficient plant viral vectors. Within this order, the softbodied aphids (Family Aphididae) account for more than 50% of the vector species that transmit over 60% of the viruses [55]. Over 20 different aphid species are able to transmit MDMV non-persistently [56]. The broad range includes *Rhopalosiphum maidis* (Fitch) (Figure 1), *Myzus persicae* (Sulzer), *Rhopalosiphum padi* (Linnaeus), *Rhopalosiphum poae* (Gill.), *Brevicoryne brassicae* (Linnaeus) and *Rhopalosiphum fitchii* (Sand.) [57].

MDMV has a short acquisition access period (AAP) of 10–30 s [58]. Aphid transmission of the virus is closely correlated to the retention of the virus in stylets [59]. Berger et al. [60] also discovered that retention of MDMV was much longer when increasing the time of acquisition time. Previously, periods of retention around 15–20 min have been recorded [58,61]. However, longer periods of up to 240 min have also been recorded by for the retention of MDMV-A by *M. persicae* [62].

**Figure 2:**
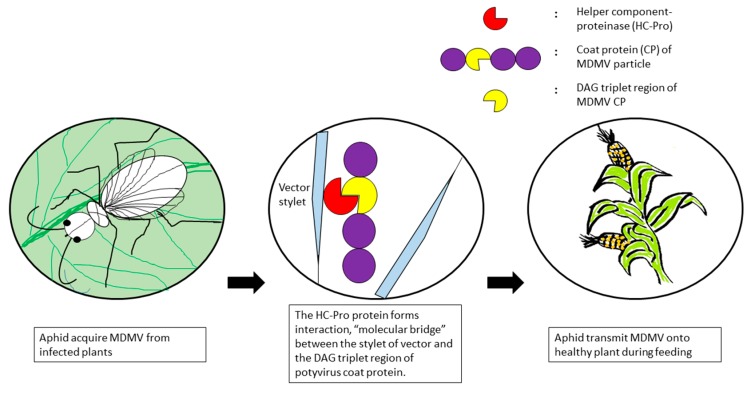
Interaction between MDMV and the vector appendage.

Appendages of aphids are where the virus particles have been retained either directly or indirectly before being inoculated into plants. There are two viral encoded factors that mediated the attachment of viruses to the appendages of aphids in indirect binding, the CP as the component of virion and the helper component-proteinase (HC-Pro) [63]. Helper component is a non-structural protein found in diseased plants but not in healthy tissues. The HC-Pro protein forms interactions between the stylet of vector and the virus coat protein, thus performs its function as a “molecular bridge” (Figure 2) [64,65]. Transmission of potyviruses is linked to a triplet 3-amino acid sequence, DAG (Asp–Ala–Gly), near the N-terminal region of the coat protein [66]. The simplest hypothesis to explain the behavioural difference of the N-terminus of the CP when it acts as a part of the virus particle or as free protein is that in the entire particle, the N-terminus is not available to interact with the aphid’s stylet and so the presence of HC-Pro is needed to cause a structural change, unfolding the N-terminus of the coat protein. Since the DAG triplet in the N-terminal region of the coat protein is crucial for aphid transmission [67], substitution of any of the 3 amino acid residues or the residue after the DAG motif will minimise aphid transmission drastically, but not mechanical transmission of the virus. Although the viral factors involved in transmission are quite clear, the receptors in aphids that allow retention as well as inoculation of non-persistent viruses remain unidentified but are believed to be localised at the distal edge of the stylet bundle [68,69].

Transmission occurs at different rates according to the influence of several factors. Firstly, fasted aphids transmit potyviruses more effectively (15%) compared to non-fasted aphids (5%) [70]. Fasting allows the interfering substances, plant components, to be egested or swallowed, clearing the alimentary canal, and hence enhances the virions retention in aphid’s stylet [71]. Furthermore, transmission of MDMV was positively related to leaf age, as the MDMV concentration was lower in older leaves, resulting in decreased aphid transmission [70]. It was found that a group of aphids improved MDMV transmission compared to single aphid transfer. This is probably due to the relatively short retention period and low transmission rate of MDMV. In addition, the efficiency of aphid transmission of MDMV is generally related to virus concentration in vivo in corn leaves. Therefore, any factors such as nutrition, host species, temperature, age of infection that could affect MDMV concentration in vivo in corn most likely would affect the efficiency of aphid transmission directly or indirectly [70]. It was also reported that the aphid species and virus strains greatly affect the efficiency of transmission [72]. Last but not least, both temperature and humidity perform an important function in the epidemiological study of MDMV. These factors greatly affect the efficiency and quantity of aphid vectors. For an example, *S. graminum* developed high populations under low temperature and low humidity early in the season, resulting in massive dispersal of viruliferous adults during the corn growing season [73].

Besides aphid transmission, MDMV can also be seed transmitted [20]. Seed transmission rates of up to 0.5% have been reported for the incidence of maize dwarf mosaic disease by Boothroyd et al. [74], and its expression appeared to be in two ways: as mosaic symptoms in certain plants and no symptoms in other infected plants. MDMV was present in 0.4% of field corn and sweet corn seeds [46] as well as in 0.2% of the tested maize inbred lines [75], both figures are based on symptom expression. Further reported rates for seed transmission in corn are 0.007% [76], 0.006% [77], 0.005% and non-existent [78]. For example, seeds dissected at different maturity stages and analysed for the presence of MDMV viruses through enzyme-linked immunosorbent assay (ELISA) and infectivity tests showed that at 21 days after pollination, MDMV was always discovered in the pericarp, but unfrequently in the endosperm or embryo [78]. The absence of virus infection in the embryo indicated limited or no seed transmission. The proportion of MDMV in seed parts declined from 100% at 21 days after pollination to rare at maturity in the pericarp. Upon reaching maturity, the pericarp transformed from a very moist nature to a hard, dry membrane. Thus, it is not unexpected since the virus within might have been inactivated. Infecting female gametophytes appeared to be the major MDMV seed transmission mechanism in sweet corn. MDMV was not detected in the male gametophytes, but was present in the unfertilized kernels, silk, glumes and whole anthers [77].

## 4. Symptom

Symptomatology of *Maize dwarf mosaic virus* has been described in several studies. In general, field plants infected by MDMV exhibit mosaic patterns [79], which normally initiate near the lower part of the youngest leaves and appear uneven and diffuse [18]. Mosaics formed by MDMV-A usually occur between leaf veins and so develop stripes [72]. Mosaics might develop as yellowish streaks that run throughout the edge of the leaf (Figure 1), and may disappear during hot weather and be replaced with common chlorosis in subsequent growth [18]. Chlorotic bands, or an “A” shape, occur when the chlorotic regions combine forming continuous streaks along the veins. Older plants develop only chlorotic indications on upper leaves and red streaks at times on mature leaves in late infections [73]. Other symptoms include mottling spots and irregular necrotic lesions [80]. During progression of earlier symptoms, dark and light green mottles appear on leaves. Formation of mosaics, flecks and rings on leaves is a consequence of an increase in intensity of dark and light green mottles as the disease develops [72]. Infected plant cells have been observed to consist of characteristic inclusion bodies, also known as cytoplasmic inclusions (CI), with closely associated cytoplasmic vesicles [81,82]. The CIs are composed of a virus encoded protein subunit with a relative molecular mass of 68 kDa [83].

Overall, infection at juvenile growth stages delays maturity and causes the loss of a large number of kernels at the basal end of the ear, which is usually known as butt blanking [23,24,84]. Occurrence of butt blanking is due to growth retardation of pollen germ tubes on diseased plants’ silks [85]. Stunting, reduction in plant weight [86], delay in silking up to four days [87], decrease in ear weight, ear diameter [88], head size and head numbers are also a part of MDMV symptomology. At times, corn plants infected with MDMV might show a delay in flowering as well as a poor grain set and fill [89].

## 5. Diagnostic Method

It is quite challenging to detect virus disease in corn by observing the symptoms alone, since symptoms vary according to plant genotype, infection time, condition of environment, and the potentiality for various infections. In consequence, observable diagnosis is validated using serological and molecular tests (Table 2). Available diagnostics for MDMV include commercialised enzyme-linked immunosorbent assays (ELISA) using antisera and reverse transcription-polymerase chain reaction (RT-PCR) involving virus genome sequence [3].

It is vital for the identification of MDMV to be confirmed using RT-PCR or ELISA. The advantage of RT-PCR is that it is much easier to acquire sequences from the database and thus information about the virus strain and basis of the viral isolates can be gathered [18]. RT-PCR procedure for MDMV strains begins with the grinding of maize infected sample in suitable extraction buffer, such as basic phenol or detergent mixture, followed by addition of ammonium acetate and chloroform-isoamyl alcohol and then centrifugation at low speed to obtain the nucleic acid containing supernatant [90]. Precipitate of the supernatant will be washed using 70% ethanol and resuspended, from which one microlitre of total RNA will be proceeded to the first cDNA synthesis [30]. This will be carried forward in order to perform RT-PCR using primers specifically designed for amplification of the available MDMV genome sequence. RT-PCR product will be sequenced and compared with known MDMV sequences. This will be carried out by conducting multiple sequence alignment (MSA). A previous study by Giolitti et al. [19] reported that the virus infecting maize fields in Chile is closely related to MDMV isolate from Argentina (MDMV-Arg), since RT-PCR conducted on those infected maize sample from Chile using primers designated for the gene MDMV-Arg capsid protein yielded only one band of the size, as expected.

For the serology part, the principle involved is simple. When an antigen is injected parenterally into the body of a rabbit followed by booster injections, it will trigger the release of particular antibodies. Rabbit will be then bled to obtain the blood serum containing immunoglobulins [88]. The antibodies respond to the antigen that activated their formation specifically in some observable ways. Plant viruses could be used as antigens, and in any plant suspected to have infection the presence of that particular antigen can be determined by using homologous antisera [98]. The ELISA method of serology is a hypersensitive technique for diagnosis of lower antigen concentrations in both crude and purified extract of viruses [99]. Its specificity is also useful for the differentiation of very closely related viral isolates [100]. ELISA tests are extremely cost effective as well as being relatively to use on an easy routine basis.

Although many variations have been developed, most investigators favour the ELISA type called double-antibody sandwich (DAS). A capture antibody is employed to coat a solid phase and is used to immobilize the virus in this assay. A second antibody, conjugated with an enzyme generally alkaline phosphatase is used to detect the immobilized virus by virtue of reaction with a substrate appropriate to the enzyme. The polyclonal antisera utilized in this assay is also often able to respond to antigens from uninfected plant extracts, since the avidity and affinity of the antibody molecule for the virus has been altered, which induced modification in their strain specificity [96]. This is due to the conformational changes in the polyclonal antibody molecule caused by conjugation of the enzyme to the second antibody [101,102]. This problem can often be circumvented by using capture and second antibody prepared in different animal species and detecting the second antibody with an enzyme-conjugated anti-species specific antibody [103]. However, specific antisera from different animal species are often unavailable. The unique characteristics of monoclonal antibodies offer the potential to develop a DAS-ELISA that avoids modifications of second antibody reactivity by molecular conjugation and does not require use of antisera raised in different animal species [92]. Almost all the monoclonal antibodies are specific to the homologous antigen. Utilization of monoclonal antisera for the detection of plant virus in diagnosis and epidemiology is a possible replacement for the use of polyclonal antibodies [96].

For this DAS-ELISA assay, results will be interpreted from the absorbance readings at 405 nm apart from examining the wells visually or with the aid of a plate reader for any colour change. Development of yellow colour in wells shows positive reactions, whilst negative reactions are demonstrated by no critical development of colour in the wells. Test values are considered to be valid if, and only if, wells of positive control result in a positive reaction while the healthy control and buffer containing wells stay clear. Samples of extract that give absorbance readings greater than two times the average of healthy controls could be read as a positive result [18]. Generally, for the positive results, similar values should be read in comparison of A405 nm values of the infected leaves sap to that with purified MDMV.

ELISA was carried out on hundreds of MDMV infected and non-infected corn plant saps, with a 100% correct result of the positive and negative controls. The sensitivity of ELISA test is 100 times higher compared to the regular tests of infectivity. Hence, application of ELISA for the diagnosis of plant viruses is much encouraged. This is also because the visual evaluation of the assay on the presence of viruses in plant tissue extracts is found to be reliable [92].

## 6. Control

### 6.1. Use of Insecticide and Johnsongrass Eradication

Once the presence of MDMV in plant has been detected, it is important to implement control strategies to reduce further yield loss. Arthropods play a role as a transmitting agent for all economically vital virus-induced diseases, including MDM disease in corn. Hence, occurrence of the disease requires three elements to be present simultaneously in a relevant environment: the virus, the transmitting vector and a vulnerable host [3]. One of the common approaches to control the spread of virus diseases in corn is by interrupting vector-maize interaction by reducing vector numbers on susceptible maize. This is possible by applying chemical insecticides or aphicides [104]. However, this method only restricts internal spread of the virus within a site and unfortunately affect the soil fertility [105]. Furthermore, previously Toler [106] demonstrated that MDM disease is relatively unaffected by the use of insecticides. Another common measure to control maize dwarf mosaic is breaking pathogen-vector and pathogen-maize interrelations by eliminating virus sources [3]. Johnsongrass, *Sorghum halepense*, is the main host of MDMV [107,108]. Yossen et al. [109] identified an MDMV isolate from johnsongrass and the plant can act as an overwintering host providing a shield for the virus [19]. Similarly, infection of MDMV in Spain is reported to be correlated with the abundance of johnsongrass [110]. Therefore, eradication of johnsongrass could effectively limit virus spread [111]. However, it is extremely difficult to do this in fields continuously cropped with maize. Thus, it is advisable to carry out crop rotation with a non-gramineous crop like soy bean, *Glycine max*, in fields with a perennial johnsongrass problem or a history of maize virus diseases. This has special merit with the advent of new “over-the-top” graminicides that can be used throughout the season in soybean fields to eradicate johnsongrass, with no residue carryover the following year. In addition, troublesome fields could be left fallow for half a season and an intensive effort at johnsongrass eradication could be attempted [73]. Apart from johnsongrass, the mature maize crop has the potential to act as a reservoir of virus for a newly cultivated crop, again serving as a transmitting agent source for viruses [112,113]. In this case, postponing the date of planting maize to prevent the abundancy of vector populations could allow some control over the disease.

### 6.2. S-Methylmethionine Pre-Treatment

**Figure 3:**
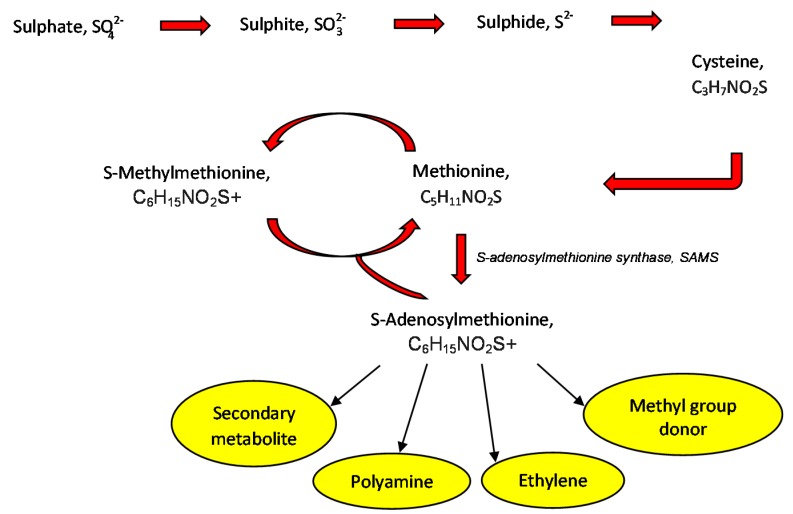
Outline of regulatory and protective compounds anabolism stimulated by S-Methylmethonine.

Plants are specified with sulphur autotrophy, a series of actions whereby sulphur is absorbed in the oxidised forms from the land, then undergo reduction before being added to cysteine and methionine. The final compound could be converted into S-Adenosylmethionine (SAM), a substrate that takes part as a methyl donor in the synthesis of S-Methylmethionine (SMM) derived from methionine [114,115]. SMM [(CH_3_)_2_-S-(CH_2_)_2_-CH(NH_2_)-COOH] naturally exists in the kingdom plantae as a non-coded and sulphur consisting amino acid [1]. SMM could be converted back into methionine through in a cyclic pathway, called the SMM cycle [116] (Figure 3).

**Figure 4:**
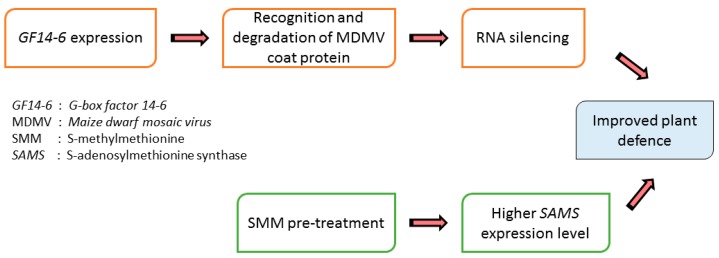
Effects of S-methylmethionine application on *GF14-6* and *S-adenosylmethionine synthase* (SAMS) expression level in MDMV infected maize plants.

SMM confers resistance in plants, being a direct precursor of osmoprotectant sulfopropionates, and it also stimulates the anabolism of other regulatory and protective compounds such as polyamine and ethylene [117,118]. When stress factors are present, the up-regulation of the phenylpropanoid biosynthetic pathway is detected, and that contributes to the production of certain fenoloids, flavonoids and anthocyanins, which are compounds characterised by antioxidant quality and show higher absorbance in the UV spectrum [119]. The potential of SMM has been proven not only against abiotic, but also against biotic stresses, since SMM-treated plants demonstrated higher resistance against *Maize dwarf mosaic virus* infection. The expression changes of 14-3-3-like protein gene *G-box factor 14-6*, *GF14-6* and *S-adenosylmethionine synthase (SAMS)* were studied in Ludsmerszki et al. [1] during MDMV infection. The product of *GF14-6* recognises and degrades the viral coat protein, contributes to RNA silencing. In the SMM pre-treated and afterwards infected plants, a decline in the *GF14-6* expression indicates the improved plant defence due to SMM pre-treatment. Additionally, the more prolonged and long-lasting increment is measured in *SAMS* expression resulting from SMM pre-treatment followed by the infection indicates the importance of the gene product in upregulating SAM formation for methylation processes, thus raising the SMM circular pathway, and further contributing to defence compounds production [1] (Figure 4). The production of defence compounds due to SMM pre-treatment is further supported in the study by [119]; the study showed an incremental increase in the intensity of emitted fluorescence at 440 and 520 nm after plants were treated by SMM, which indicates that the amount of phenoloids increased in these plants. Kocsis et al. [120] demonstrated that during stress conditions SMM prevents chlorophyll loss. The value of the F690/F740 ratio is inversely proportional with a decrease in the amount of chlorophyll localised in the leaves, indicating the increase in chlorophyll content when the plants were treated by SMM. Similar results were achieved with chlorophyll-a fluorescence induction measurements. The Fv/Fm value, that indicates the physiological status of the plant, more specifically the maximal quantum efficiency of PSII decreased in the case of infected plants, was analysed but no significant change was detected when the infected plants also got SMM treatment prior to infection [119].

**Figure 5:**
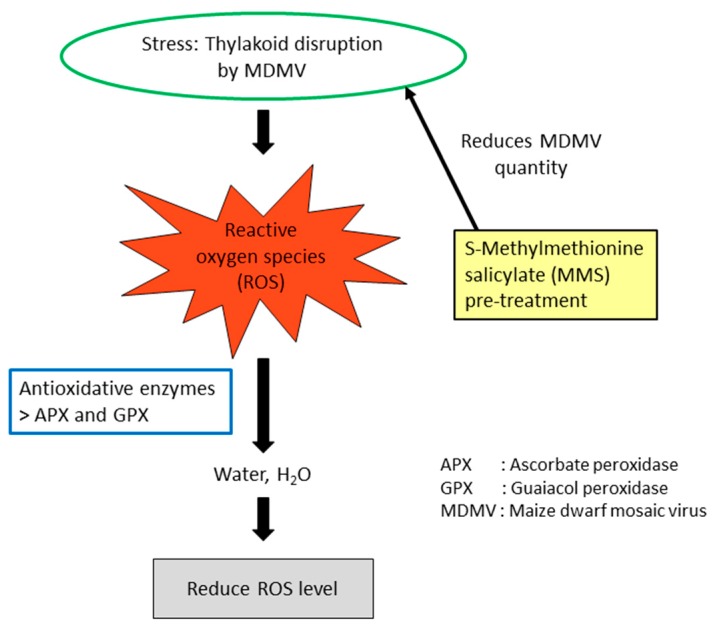
Role of S-Methylmethionine salicylate in the prevention of reactive oxygen species (ROS) development in corn plants.

Since MDMV disrupts the thylakoid [121] and any damage in thylakoid membranes causes the development of highly reactive oxygen species (ROS), this is an area of research focus. Compared to other species of plants, maize vascular bundle sheath cells are unusually sensitive to oxidative stress [122,123]. This is solved by the existence of antioxidant enzymes, primarily ascorbate peroxidase (APX) and guaiacol peroxidase (GPX) in plant cells, which protect the cell through direct scavenging of those reactive molecules [124]. When the combined molecule of SMM and salicyclic acid (SA), known as S-Methylmethionine salicylate (MMS), was applied before infection, lower levels of enzyme activity were recorded, indicating a decreased resistance level, which can be explained by the reduction in the quantity of virus particles due to MMS pre-treatment [125] (Figure 5).

During MDMV infection, the viral particles accumulate in the cytoplasm of the leaf mesophyll cells and may use chloroplasts for their replication [126,127]. The associations between virus coat protein with PSII reaction centres in thylakoid membranes lead to formation of non-fluorescent trap for excitational energy which subsequently will cause an increase in non-photochemical quenching [128]. Accordingly, Ludmerszki et al. [125] reported greater ΔpH dependent process high energy state, qE values for MDMV-infected plants which indicated the appearance of additional quenches of fluorescence in those plants. However, when infected plants were pre-treated with MMS, low qE values were measured, indicating that virus coat protein-thylakoid membrane associations were not formed. This is supported by the ELISA results, showing smaller concentration of MDMV coat protein and viral RNA in the MMS + MDMV plants, giving a clarification for the lower qE values. In general, the protecting effect of SMM treatment for maize plants prior to stress of MDMV infection is being demonstrated in clear entirely.

### 6.3. Conventional Resistance Breeding

The most effective way to protect corn crops against MDMV is through the breeding of maize lines for resistance [129,130]. Having a limited knowledge of resistant genes and the pathogens, corn breeders and co-operating pathologists with entomologists suggested that the initial step for this method is the identification of resistance sources through screening of the collected germplasm [3]. The resistance screening methods are comprised of natural infection under field conditions, vector inoculation, mechanical inoculation and growing under greenhouse conditions. 

Initially, susceptible inbred lines are crossed with resistant ones, and the F1 generation is produced. Some F1 generations are selfed to result in F2 generations, others are back-crossed with susceptible parents to produce BC1 generations [131]. Backcrosses have usually been utilized to transfer virus resistance from resistant, but agronomically undesirable, lines to well-adapted elite lines. The recurrent parent is commonly selected due to its ability to combine well, produce high yield and adapt to wider changes [132]. Selection in F2 and BC1 generations seems to be a favourable method to breed new virus-resistant inbred lines. Such selection for resistance under higher temperatures in the greenhouse normally results in the lines with higher resistance level. Accordingly, three dent inbred lines (D21, D32, FAP136OA) with complete resistance, and four dent inbreds (D06, D09, R2306, FAP1396A) with partial resistance against MDMV under both field and greenhouse conditions were determined. Apart from these, the inbred line Pa405 has been found to be the best known source of resistance to MDMV to date [133,134].

Successful screening is continued with identification of markers linked to genes or QTLs exerting resistance, which can be done using reciprocal translocations [135,136], morphological markers [137] and restriction fragment length polymorphism (RFLP) analysis [138,139]. RFLP analysis of individual back cross plants of the genotypes (Pa405 × yM14 and (Pa405 × K55) × K55, inoculated with MDMV, mapped this resistance gene to a region close to the centromere of chromosome 6. This gene is linked tightly to and located between RFLP marker loci, UMC85 and BNL6.29, and designated as gene *Mdm*1, gene 1 [140]. *Mdm*1 is crucial for any resistance reaction because all plants with missing *Mdm*1 gene rapidly developed symptoms of generalised mosaics. The resistance dominance of *Mdm*1 was further confirmed in 42 of the 43 inbred lines analysed in the study by [141].

The inheritance of resistance to maize dwarf mosaic disease is also controlled by modifiers or minor genes [142]. For an example, when F1 hybrids, F2 progeny and a recombinant inbred line (RIL) population obtained from a crossing of Oh1V1 to the virus susceptible line Oh28 were examined for their responses to six viruses including MDMV, a dominant QTL responsible for 79% of total variance and several minor QTLs each contributing to 1% of the variance mapped to chromosome 3, and 10 were identified [143]. Moreover, the data representing the percentage of infected plants denoting critical differences both within F1 generations (from 18 to 34%) and within BC1 generations (from 26 to 53%) showed that resistance to MDMV is controlled by major genes, yet the minor genes also involved in modification of the resistance [131]. These hybrids and inbred lines with genes for resistance to MDM do not show asymptomatic reactions after infections constantly [133,144]. Scott and Rosenkranz [145] mentioned that symptomatic responses maybe resulted from micro environmental influences, expression of resistance at late growth stages, concentrations of inoculum above a specific threshold. Both symptomatic tissue with high virus concentration and asymptomatic tissue with no virus detected formed distinct sectors in resistant hybrids [146]. Similarly, ELISA confirmed existence of both inoculated leaves with a positive response for virus and freshly developed leaves with no virion assembly in the same resistant inbred [147].

This indicates that MDMV can undergo replication and spread from cell to cell in leaves of infected plants, but that a barrier to systemic viral propagation is present in resistant plants. Thus, in resistant plants, the virus is restrained in its movement through the leaf vascular system [147]. The transfer of the resistance from resistant hybrids such as Pioneer Brand (PB) 3187 [148] into selected breeding lines will lead to similar resistance mechanisms and result in successful control over maize dwarf mosaic disease.

### 6.4. Genetically Engineered Resistance

Pathogen-derived resistance, which can be achieved through expression of resistant genes (Table 3), viral proteins or RNAs in transgenic plants through genetic engineering, is another strategy to curb maize dwarf mosaic disease in maize crops [149,150]. For this way of controlling, it is important to acquire knowledge on the concept of resistance expression genetically. As explained earlier, the virus resistance in maize was due to an obstruction to systemic movement of virus in resistant plants [151] where the movement could be prevented by inhibition of the loading of virions, ribonucleoprotein [152], or RNA into phloem or unloading from phloem in either leaves or roots [148]. However, for a better understanding on the mechanism for genetic resistance, global transcriptional response of resistant (Pa405) and susceptible (Oh28) inbred lines was analysed in germinating maize embryos at 4 days post inoculation (dpi). There were no groupings of differentially expressed transcripts found in the previously recognised QTLs crucial for MDMW resistance, on chromosome 6, 3 and 10 [153]. Almost 15% of transcripts were differently expressed between the resistant and susceptible lines. Of transcripts with more than 10-fold greater expression in one line, more than 70% were upregulated in the resistant line. Hsp20/alpha crystalline-like gene and cytochrome P450 are among the differentially regulated transcripts that were explained to be involved in virus resistance. The obtained responsive transcripts required to be further studied on in order to figure out their contribution for resistance [154].

Another effort of cloning *Mdm*1 allele by chromosome walking was taken to explain the biological basis of *Mdm*1 mediated resistance in resistant maize [156,157]. Although co-segregation of *Mdm*1 with the nucleolus organizer region (nor) in maize caused a difficult obstacle to clone *Mdm*1, yet this initiative still led to the achievement of a high-resolution genetic map in the region of *Mdm*1 that has raised tools for physical mapping of *Mdm*1, which is very useful for subsequent genetic engineering procedures [158].

H9-21 is one of the incorporated resistant germplasms against MDMV previously. However, it does not achieve the rate of maize production required due to its inefficient agronomic characteristics [159]. The attempts to genetically improve resistance against MDMV in maize crops were then continued with the development of engineered lines in order to generate cross protection. Cross protection can be triggered by introducing an antisense sequence homologous to MDMV genes of coat protein (CP), replicase and movement proteins that play a role in viral replication, spreading, and particle coating in the single-standard RNA viral genome [160,161]. Among these genes, maize plants transformed with MDMV protease gene (P1), a replication associated protein gene, proved to be resistant to MDMV under controlled conditions [162]. This was possible by the means of RNA interference (RNAi), mediated by self-complementary hairpin RNA (hpRNA), transcribed from a transgenic inverted-repeat sequence. RNAi has been proved to be more useful in conferring virus resistance through gene silencing due to its straight-forward natural defense mechanism [163,164,165]. MDMV resistance generated by RNA interference depends on the length of inverted-repeat sequence, the copy number of T-DNA integration and the repeatability of integration sites [166]. A longer hpRNA is more successful in the virus gene silencing rather than a shorter one [167,168]. Previously, the transfer of 150bp inverted-repeat sequence homologous to MDMV P1 enhanced maize virus resistance yet resulted in T2 lines with intermediate resistance or lower compared to the resistant control. Then, MDMV resistance was further improved through development of an hpRNA expression vector consisting of 451bp inverted-repeat sequences of MDMV protease gene (P1) [169]. Generally, genetic improvement of maize is considered to be a more efficient and more environmentally sustainable measure for virus management [87,170].

## 7. Concluding Remarks

Detailed studies conducted on the genome and symptomatology of MDMV have resulted in successful visual, serological, as well as molecular, tests of diagnosis. This contributes to efforts of controlling MDM disease in order to reduce the yield loss caused in corn crops. The methods that were utilized in circumventing this problem include eradication of johnsongrass, SMM pre-treatment, resistance breeding and genetic engineering. The efficiency of currently available measures for curbing the spread of MDM disease has been proved, shown by the now rare infection of MDMV being observed in field corn. However, further initiatives can be focused on understanding of maize resistant genes using omics technology approach against MDMV for a better management of MDM disease of maize worldwide.

## Figures and Tables

**Table 1:** Available complete sequences of *maize dwarf mosaic virus* (MDMV) isolates.

MDMV Isolates	Geographical Origin	Genbank Accession	References
MDMV	Golestan	JQ280313	[38]
MDMV-Bg	Bulgaria	NC003377	[27]
MDMV-Sp	Spain	AJ416645	[39]
MDMV-OH1	Ohia	JQ403608	[40]
MDMV-OH2	Ohia	JQ403609	[40]
MDMV-It	Italy	JX185302	[41]

**Table 2:** Methods for *Maize dwarf mosaic virus* (MDMV) detection.

Method	Platform	Effect on MDMV Variation	Reliability	Efficiency	References
Indirect Enzyme-Linked Immunosorbent Assay (ELISA)	Rabbit anti-mouse IgM capture monoclonal antibody Rabbit anti-mouse IgG second monoclonal antibody	MDMV-A and MDMV-B	Allow detection of MDMV-A and MDMV-B bound IgG among tested leaves sap infected with several different strains of MDMV and *Sugarcane mosaic virus* (SCMV).	MDMV-A-specific ELISA = antigen detected in sap at a dilution of 1:60 with optimal sample pH (7.5–8.5). MDMV-B-specific ELISA = detect antigen in sap at dilution end point of 1:2560	[91]
Indirect Enzyme-Linked Immunosorbent Assay (ELISA)	M-C antiserum M-D antiserum	M-C and M-D	M-C antigen reacts strongly with M-A, M-D, *Sorghum red stripe virus* (SRV) antisera. M-D antigen reacts strongly with M-D, M-C antisera.	M-C particles react with M-A and SRV antisera in the range of 1:128 to 1:512 dilution end points, with M-D antisera at dilution end point of 1:4. M-D antigen reacted with homologous antisera, M-D up to a dilution of 1:512, with M-C antisera at 1:16 dilution.	[88]
Double-Antibody Sandwich Enzyme-Linked Immunosorbent Assay (DAS ELISA)	Anti-MDMV-A rabbit serum purified for IgG Alkaline phosphatase coupled to purified IgG as conjugate	MDMV-A	Visual evaluation at absorbance of 405 nm gives reliable information on MDMV-A presence in leaves extracts tested.	Visible yellow colour formed in wells with antigen diluted up to 10^−4^. Sensitivity is 100 times better than conventional infectivity test.	[92]
Double-Antibody Sandwich Enzyme-Linked Immunosorbent Assay (DAS ELISA)	IgG purified from polyclonal anti-MDMV serum Alkaline phosphatase coupled to purified IgG-E as conjugate	MDMV-A, MVMV-J, MDMV-L, MDMV-SP, MDMV-YU	Among all reference strains, only MDMV strains react positively in DAS-ELISA (recorded OD 405 nm values at least twice the healthy sap OD 405 nm values) with anti-MDMV IgG.	IgG antibody dilution to 1 μg/mL able to detect MDMV antigen dilution to 1/100.	[30]
Double-Antibody Sandwich Enzyme-Linked Immunosorbent Assay (DAS ELISA)	MDMV-Arg (Argentina strain) [93] specific polyclonal IgG Alkaline phosphatase-conjugated IgG as secondary antibody	MDMV	DAS-ELISA absorbance values of infected maize leaf samples from field grown plants and maize leaf sample with MDMV-Arg isolate were highly significant while the values from healthy control and buffer were very low.	MDMV-Arg specific polyclonal IgG strongly reactive up to 1:2000 dilution with MDMV antigen from infected field, Chile.	[19]
Capture Reverse Transcription-Polymerase Chain Reaction (RT-PCR)	Primers designed to MDMV-Arg [93] capsid protein gene	MDMV	Single band of expected size (1104 bp) obtained.	Detect MDMV samples from Chile which closely related to MDMV-Arg (Argentina strain) efficiently.	[19]
Reverse Transcription-Polymerase Chain Reaction (RT-PCR)	Fwd primer = oligo1n: ATGGTHTGGTGYATHGARAAYGG Rvs primer = oligo2n: TGCTGCKGCYTTCATYTG *single lettercode: H = A/C/T, Y = C/T R = A/G, K = G/T	MDMV-SP, MDMV-Bu, MDMVJIL	Single product of expected size (327 nts) obtained for all tested MDMV isolates.	Efficient for dealing with well-characterized strains, field collected isolates.	[30]
Reverse Transcription-Polymerase Chain Reaction (RT-PCR)	Universal primer (Sprimer: 5′-GGXAAYAAYAGYGGXCAZCC-3′, X = A/G/C/T; Y = T/C; Z = A/G) M4 primer	MDMV	cDNA fragments of expected size were amplified from the 3′ terminus of RNA genomes of 21 different viruses under family *potyviridae* including MDMV.	Universal primer designed based on an alignment of the amino acid sequences around the conserved GNNSGQP motif in nuclear inclusion body b (NIb) gene of family *potyviridae* members. Hence, it is proved useful for detection of *potyviridae* members.	[94]
Reverse Transcription-Polymerase Chain Reaction (RT-PCR)	MDMV F1: 5′-CAACCAGGGCYGAATTTGATAG-3′ MDMV R1: 5′-GTGCAAGGC TRAAGTCGG TTA-3′	MDMV	Supposed to yield a PCR product of expected size (336 bp)	MDMV can be distinguishable from *Sugarcane mosaic virus*, SCMV and *Johnsongrass mosaic virus*, JGMV through RT-PCR	[57]
Combined Reverse Transcription Polymerase Chain Reaction (RT-PCR) with Electrochemiluminescence method	Specific nucleic acid sequences (20 bp) were added to 5′ terminal of all primers Biotin was introduced into reverse primer	MDMV	PCR yielded a product with a single band of expected size (643 bp) for all 4 tested viruses including MDMV.	This method has higher sensitivity and lower cost than others. It can effectively detect the MDMV with simplicity and stability.	[95]
Competitive Radioimmunoassay (RIA)	Rabbit anti-mouse monoclonal IgG	MDMV-A (referred here as MDMV-AP) and MDMV-B	Feasible alternative to the use of polyclonal antisera in detecting homologous viruses (MDMV, *Soybean mosaic*, SMV, *Lettuce mosaic virus*, LMV).	Antigen (purified virus) detected at dilution of 10–50 ng/mL.	[96]
Dot Blot Immunoassay	MDMV-Arg [93] polyclonal antiserum	MDMV	MDMV symptomatic field grown plants had strong reaction with the polyclonal antiserum against MDMV-Arg isolate while healthy plants were negative.	MDMV samples reacted with MDMV-Arg polyclonal antiserum of dilution up to 1:5000	[19]
Sodium dodecyl sulfate (SDS) immunodiffusion test	M-D antiserum M-D antiserum	MDMV-C and MDMV-D	M-C antiserum reacted with both M-C and M-D antigens forming a spur, which indicate partial serological relatedness. M-D antiserum reacted with its homologous viral antigen, M-D.	No precipitin lines were obtained when antisera reacted against healthy crude sap	[88]
DNA Microarray (Maizepath)-based detection	Microarray with 60-mer oligonucleotide probes complementary to genomes of 5 maize pathogens including MDMV	MDMV	Obtained results indicate that the fluorescence signals from MDMV, other pathogens and control probes are well distinguished in all performed experiments.	Gives more than 180 K probes in total, thereby classified as high-density microarray that able to investigate thousands of genomic loci in a high-resolution manner.	[97]

**Table 3:** Summary on genes conferring resistance against *maize dwarf mosaic virus* (MDMV).

Chromosome	Locus	Resistance Source	Screening Method	Level of Resistance	References
6 (short arm)	*Mdm*1	Pa405 Oh1V1	Mechanical inoculation/Greenhouse Mechanical inoculation/Field	High resistance (Dominant gene)	[140,143]
6	*Mdm*1 co-localizes with *Wsm*1	*Wsm*1 NIL (near isogenic lines) Oh28^SS/RR/SS^ Left: *Wsm*2 alleles Middle: *Mdm*1/*Wsm*1 alleles Right: *Wsm*3 (Two *Mdm*1/*Wsm*1 allele)	Mechanical inoculation/Greenhouse/Field condition	High resistance	[80]
6	*Mdm*1 co-localizes with *Wsm*1	*Wsm*1 × Oh28 F1 Oh28^SS/RS/SS^ Left: *Wsm*2 alleles Middle: *Mdm*1/*Wsm*1 alleles Right: *Wsm*3 (One *Mdm*1/*Wsm*1 allele)	Mechanical inoculation/Greenhouse/Field condition	Intermediate resistance	[80]
3 & 6	*Wsm*2 combine with *Mdm*1/*Wsm*1	*Wsm*1 *Wsm*2 NIL Oh28^RR/RR/SS^ Left: *Wsm*2 alleles Middle: *Mdm*1/*Wsm*1 alleles Right: *Wsm*3	Mechanical inoculation/Greenhouse/Field condition	Lowers symptom incidence	[80,142]
3 & 6	*Wsm*2 combine with *Mdm*1/*Wsm*1	*Wsm*1 *Wsm*2 × Oh28 F1 Oh28^RS/RS/SS^ Left: *Wsm*2 alleles Middle: *Mdm*1/*Wsm*1 alleles Right: *Wsm*3	Mechanical inoculation/Greenhouse/Field condition	Lowers incidence and severity of disease	[80,142]
6 & 10	*Wsm*3 combine with *Mdm*1/*Wsm*1	*Wsm*1 *Wsm*3 × Oh28 F1 Oh28^SS/RS/RS^ Left: *Wsm*2 alleles Middle: *Mdm*1/*Wsm*1 alleles Right: *Wsm*3	Mechanical inoculation/Greenhouse/Field condition	Lowers disease incidence and severity	[80,141]
3 & 6	*Scmv*2 combined with *Scmv*1	F7 ^RR/RR^ Left: *Scmv*2 alleles Right: *Scmv*1 alleles R: Susceptible parent F7 R: Resistant parent FAP1360A	Mechanical inoculation by rubbing infected leaves	Complete resistance	[155]
